# Supplementary material for: Enhanced thermally-activated skyrmion diffusion with tunable effective gyrotropic force
Source: Nat Commun. 2023 Sep 11;14:5424. doi: 10.1038/s41467-023-40720-0 (PMC10495465; doi:10.1038/s41467-023-40720-0)
Supplement: Supplementary file 1 — Supplementary Information [file 41467_2023_40720_MOESM1_ESM.pdf]

Enhanced thermally-activated skyrmion diffusion with tunable effective gyrotropic force

Takaaki Dohi[1, 2]*, Markus Weißenhofer[3, 4, 5]*, Nico Kerber[1, 6], Fabian Kammerbauer[1], Yuqing Ge[1], Klaus Raab[1], Jakub Zázvorka[7], Maria-Andromachi Syskaki[1, 8], Aga Shahee[1], Moritz Ruhwedel[9], Tobias Böttcher[6, 9], Philipp Pirro[9], Gerhard Jakob[1, 6], Ulrich Nowak[3], and Mathias Kläui[1, 6]*

[1] Institut für Physik, Johannes Gutenberg-Universität Mainz, Staudingerweg 7, 55128 Mainz, Germany

[2] Laboratory for Nanoelectronics and Spintronics, Research Institute of Electrical Communication, Tohoku University, Sendai 980–8577, Japan, Japan

[3] Fachbereich Physik, Universität Konstanz, DE-78457 Konstanz, Germany

[4] Department of Physics and Astronomy, Uppsala University, P.O. Box 516, S-751 20 Uppsala, Sweden

[5] Department of Physics, Freie Universität Berlin, Arnimallee 14, D-14195 Berlin, Germany

[6] Graduate School of Excellence Materials Science in Mainz, Staudingerweg 9, 55128 Mainz, Germany

[7] Institute of Physics, Faculty of Mathematics and Physics, Charles University, Ke Karlovu 5, Prague 12116, Czech Republic

[8] Singulus Technologies AG, 63796 Kahl am Main, Germany

[9] Fachbereich Physik and Landesforschungszentrum OPTIMAS, Technische Universität Kaiserslautern, Gottlieb-Daimler-Straße 46, 67663 Kaiserslautern, Germany

E-mail addresses

Dr. Takaaki Dohi: tdohi@tohoku.ac.jp

Dr. Markus Weißenhofer: markus.weissenhofer@uni-konstanz.de

Prof. Dr. Mathias Kläui: klaeui@uni-mainz.de

SUPPLEMENTARY INFORMATION

Note 1. Interlayer thickness dependence of magnetization curve

Note 2. Intensity comparison of Kerr rotation angle

Note 3. Temperature dependence of interlayer exchange coupling

Note 4. Rigid body description of skyrmion motion in synthetic antiferromagnets

Note 5. Size evaluation of skyrmions

Note 6. Quantitative evaluation of interfacial Dzyaloshinskii-Moriya interaction

Note 7. Chirality evaluation for bi-layer systems

Note 1

Interlayer thickness dependence of magnetization curve

In order to investigate the sign and strength of the interlayer exchange coupling, we investigate the Ir thickness t_{Ir} dependence of the magnetization curve using the Kerr microscope in the polar configuration. Supplementary Fig. 1a shows the stack structure used for investigating the t_{Ir} dependence where t_{Ir} ranges from 0.4 to 1.5 nm in steps of 0.1 nm. In order to unambiguously obtain the second peak of the interlayer exchange coupling oscillation, a stack with a large effective perpendicular anisotropy energy density ($K_{eff} > -J_{int}/t_T$) was used.

As shown in supplementary Fig. 1b, the stack with t_{Ir} = 1.2 nm shows the second peak of the interlayer exchange coupling field $\mu_0 H_{int}$ defined by a magnetic field that induces the spin flip-like antiferromagnetic (AFM)-ferromagnetic (FM) transition, which is consistent with previous work[1]. Based on this experimental result, we chose t_{Ir} to be 1.2 nm for this work in the main text. Possibly, one could use the first peak of the oscillation corresponding to t_{Ir} = 0.5-0.6 nm which exhibits much larger interlayer exchange coupling. However, such a thin Ir layer causes a large difference in magnetic properties such as K_{eff} between the top and bottom ferromagnets since we need a relatively thick Pt layer (at least 1 nm) to make the entire system exhibit a perpendicular easy axis. The large difference presumably produces an additional contribution to pinning. Therefore, we opted to use the second peak rather than the first peak.

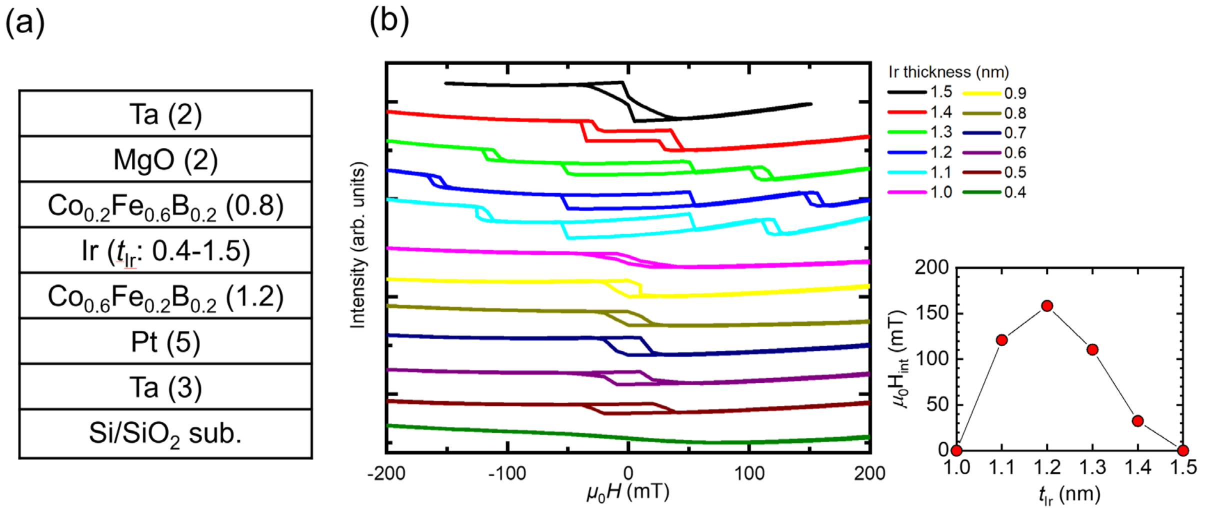

Supplementary Figure 1 | Ir thickness dependence of Kerr hysteresis curve. **a**, Stack structure employed for investigating the Ir thickness dependence of the interlayer exchange coupling. The unit of the number shown in parentheses is a nanometer (nm). **b**, Ir thickness dependence of the Kerr hysteresis loops and the oscillation of interlayer exchange coupling field $\mu_0 H_{\text{int}}$.

Note 2
Intensity comparison of Kerr rotation angle

To independently confirm the strong compensation of the two layers, we compare the Kerr intensity for the FM bi-layer and SyAFM systems at room temperature using the same conditions. Supplementary Fig. 2 shows the intensity comparison of the Kerr rotation angle. We clearly see the conspicuous decrease of the Kerr rotation angle for the SyAFM system with 90% compensation, which is independent evidence of the AFM coupling. Additionally, we can barely detect the switching of the magnetization for a more compensated state (indicated by “More than 90%”). The contrast for the domain state imaging becomes too low, which makes the domain and skyrmion tracking challenging albeit image processing. Thus, we designed the maximum compensation as 90% for our analysis.

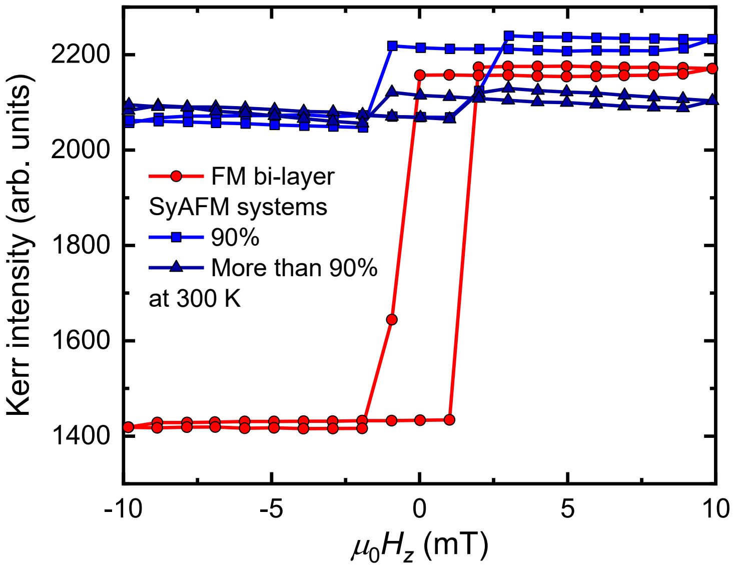

Supplementary Figure 2 | Intensity comparison of the Kerr rotation angle for the FM bi-layer and SyAFM systems at room temperature.

Note 3
Temperature dependence of interlayer exchange coupling

Here we investigate the temperature dependence of the interlayer exchange coupling to ascertain if there is the possibility that the AFM coupling vanishes with temperature. Hall measurements using the Van der Pauw method were employed for high accuracy measurements. Supplementary Fig. 3a and b show the transverse resistance R_{xy} along the current flow direction as a function of out-of-plane magnetic fields, and its derivative, respectively. Based on the peak indicating the interlayer exchange coupling field in supplementary Fig. 3b, we deduce that the change of coupling strength is less than 10 mT (corresponding to the step of $\mu_0 H$) for a 10 K temperature change. In general, the temperature dependence of interlayer exchange coupling is expressed as an effective power-law[2],

$$f(T) = 1 - xT^n, \quad \text{(S1)}$$

where n can be roughly approximated as 1.5 on the basis of the experimental observation[3–6]. Hence, within the range of temperatures used in this work, the change of coupling strength is less than approximately 10 mT for all the stacks as well, meaning that the decoupling of SyAFM skyrmions does not occur for the entire temperature range studied. Also, note that the decoupling or the difference in domain size between FMs leads to a drastic enhancement of the Kerr intensity, which has never been observed in our system (at least, the size difference and its time scale is less than the resolution of a Kerr microscope), indicating the strong enough coupling against thermal agitation.

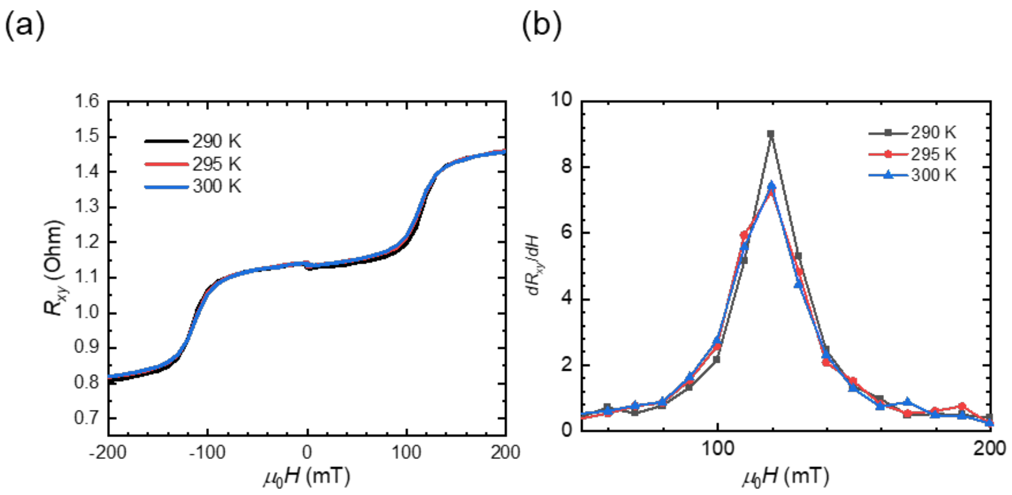

Supplementary Figure 3 | Hall measurement for the temperature dependence of the interlayer exchange coupling. **a**, Transverse resistance R_{xy} as a function of out-of-plane magnetic fields for three temperatures (black: 290K, red; 295 K, and blue; 300K.). **b**, Derivative of Fig. 3a. The peak corresponds to the strength of the interlayer exchange coupling field.

Note 4

Rigid body description of skyrmion motion in synthetic antiferromagnets

To investigate theoretically the influence of the compensation of the SyAFM system on the thermal dynamics of skyrmions, we employ a rigid body description. As such, the dynamics are described by two degrees of freedom, the position of the skyrmion, instead of $2N$ spin degrees of freedom. This greatly reduces the complexity of the problem and allows for an analytical calculation of the diffusion constant in the absence of pinning.

The rigid body description of the dynamics of localized magnetic textures was first established by Thiele[7] and relies on the ansatz $\mathbf{S}(\mathbf{r}, t) = \mathbf{S}(\mathbf{r} - \mathbf{R}(t))$. Applying this ansatz to the stochastic Landau-Lifshitz-Gilbert equation (see the Materials and Methods) yields for an FM skyrmion the following equation of motion,

$$\mathbf{G} \times \mathbf{v} + \alpha \Gamma \mathbf{v} = \mathbf{F}^{\mathrm{th}}. \qquad \text{(S2)}$$

This equation was termed Thiele equation and has proven to be quite successful in predicting the dynamics of FM skyrmions[8]. The first term is the topology-dependent gyrocoupling term and the second term describes friction of the skyrmion. The thermal force $\mathbf{F}^{th}$ was first added by Schütte et.al.[9] It has a zero mean and its autocorrelation is given by $\langle F_\mu^{th}(t) F_\nu^{th}(t') \rangle = 2k_{\mathrm{B}} T \alpha \Gamma \delta_{\mu\nu} \delta(t - t')$. In a FM bi-layer system with saturation magnetizations $|M_1|$ and $|M_2|$ the parameters in the Thiele equation read

$$\Gamma = \frac{|M_1| + |M_2|}{2\gamma} \int (\partial_x \mathbf{S})^2 + \left(\partial_y \mathbf{S}\right)^2 dxdy \text{ and } \mathbf{G} = \frac{|M_1| + |M_2|}{\gamma} 4\pi Q \mathbf{e}_z. \qquad \text{(S3)}$$

In the above expression, $Q = 1/(4\pi) \int \mathbf{S} \cdot (\partial_x \mathbf{S} \times \partial_y \mathbf{S}) dxdy$ is the topological charge of the skyrmion.

The MSD predicted by Eq. (S2) scales linearly on time and the slope is given by two times the diffusion constant, which reads[9]

$$D_{\mathrm{dif}}^{\mathrm{FM}} = k_{\mathrm{B}}T\frac{\alpha\Gamma}{\alpha^2\Gamma^2 + G^2}. \qquad \text{(S4)}$$

The validity of this expression in the limit of large values of the Gilbert damping parameter and low temperatures has been demonstrated in preceding works[10,11]. Contributions from the coupling to the magnonic heat bath that were predicted by a recent study[12] are neglected here, because they do not have a qualitative impact on the dynamics.

The effective equation of motion describing the dynamics of AFM skyrmions differs from Eq. (S2). It was demonstrated that instead they are described by a massive equation of motion that lacks a gyrocoupling term[13]:

$$M\frac{\partial \mathbf{v}}{\partial t} + \alpha\Gamma\mathbf{v} = \mathbf{F}^{\mathrm{th}}. \qquad \text{(S5)}$$

M is here the mass of the AFM skyrmion and leads to a non-linear dependence of the MSD at low times. The diffusion constant, i.e. the half of the slope of the MSD in the long time limit, however, is not impacted by the finite mass, and it follows

$$D_{\mathrm{dif}}^{\mathrm{AFM}} = \frac{k_{\mathrm{B}}T}{\alpha\Gamma}. \qquad \text{(S6)}$$

This expression was derived in Ref. 14 and its validity in atomistic spin simulations was demonstrated therein.

Here, we want to establish an effective description for the dynamics of skyrmions in SyAFMs. This is done in a heuristic manner and leads to the derivation of Eq. (1), whose validity is demonstrated using atomistic spin simulations. Depending on the compensation $m_{\mathrm{Com}} = 1 - |M_1 + M_2|/(|M_1| + |M_2|)$ of the bi-layer system, the system can be varied from a

FM to an AFM one. Likewise, the description of the dynamics of the skyrmions in the system transforms from Eq. (S2) to Eq. (S5). We make the ansatz that this transformation is linear in m_{Com} and, henceforth, the effective equation of motion for a SyAFM skyrmion reads

$$m_{\mathrm{Com}} M \frac{\partial \mathbf{v}}{\partial t} + (1 - m_{\mathrm{Com}}) \mathbf{G} \times \mathbf{v} + \alpha \Gamma \mathbf{v} = \mathbf{F}^{\mathrm{th}} \qquad \text{(S7)}$$

with **G** and Γ as in Eq. (S3). For an FM bi-layer, we have $m_{\mathrm{Com}} = 0$ and thus the mass term vanishes and the prefactor to the gyrocoupling term becomes one. For an AFM bi-layer, we have $m_{\mathrm{Com}} = 1$ and the gyrocoupling term vanishes. As such, the compensation changes the effective topological charge of the skyrmion. The impact of the varying compensation on the diffusive motion is twofold: first, it impacts the timescale of the non-linear MSD for short times, and second, it impacts the diffusion constant via

$$D_{\mathrm{dif}}^{\mathrm{SyAFM}} = k_{\mathrm{B}} T \frac{\alpha \Gamma}{\alpha^2 \Gamma^2 + G^2 (1 - m_{\mathrm{Com}})^2}. \qquad \text{(S8)}$$

Again, the limits $m_{\mathrm{Com}} = 0$ and $m_{\mathrm{Com}} = 1$ reproduce the formula for the FM skyrmions (S4) and AFM skyrmions (S6). Eq. (S8) predicts that diffusive motion is maximal if $\alpha = |G(1 - m_{\mathrm{Com}})|/\Gamma$. This implies a shift of this maximum from $\alpha = |G|/\Gamma$ for FM skyrmion to zero for AFM skyrmions.

Note 5
Size evaluation of skyrmions

As can be seen in supplementary videos 2 and 3 as well as supplementary Fig. 4a and (b), the profile of magnetic skyrmions is not always completely circular and deforms with time. Therefore, we used the radius of gyration of its Gaussian-like profile, R_g, to evaluate the size of the skyrmion following the previous work[14],

$$R_g^2 = \frac{1}{N}\sum_{i=1}^{N}(\mathbf{r}_i - \langle\mathbf{r}\rangle)^2 \qquad \text{(S9)}$$

where $\mathbf{r}_i$ corresponds to the pixel coordinates regarding the core of the skyrmion. Also, to obtain object's actual radius R_S, we corrected the values using $R_S = \sqrt{2}R_g$ which is a relationship between the actual radius and the gyration of radius for a uniform circle. After determining R_S for each frame and skyrmion, we define the averaged radius of the skyrmion as $R_{sk.ave} \equiv \langle R_S\rangle$ taking the average over all the values. The error bar was calculated from the standard deviation. The estimated average diameter ($2R_{sk.ave}$) is shown by the blue-colored bar in supplementary Fig. 4a and b, where we confirmed that the estimated average diameter roughly matches the skyrmion size in the end.

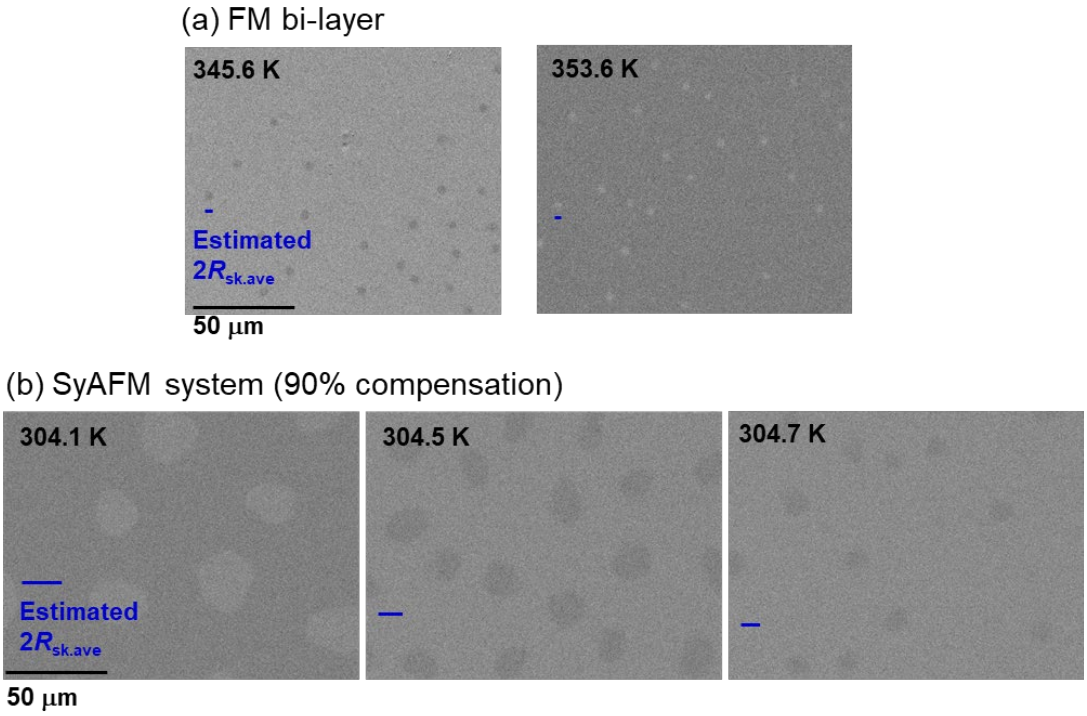

Supplementary Figure 4 | Size variation of the skyrmions. **a**, Skyrmions for FM bi-layer system at 345.6 K and 353.6 K. **b**, Skyrmions for SyAFM system with 90% compensation at 304.1, 304.5, and 304.7 K.

Note 6
Quantitative evaluation of interfacial Dzyaloshinskii-Moriya interaction

Here, we quantitively evaluate the interfacial Dzyaloshinskii-Moriya interaction and the Gilbert damping constant from measurements of the spin-wave dispersion relation. The experiments using Brillouin light scattering spectroscopy (BLS) have been conducted using a conventional, wave vector resolved BLS system in backscattering geometry using a single mode laser with λ = 491 nm wavelength. The detected spin-wave wave vector k can be selected by changing the angle of incidence θ with respect to the film normal: $k = 2\frac{2\pi}{\lambda}\sin\theta$. Data acquisition and evaluation is performed as detailed in Refs. 15 and 16.

Supplementary Fig. 5a shows the dispersion relation measured at an external field of 300 mT applied in the film plane perpendicular to the measured spin-wave wave vectors (a.k.a Damon-Eshbach geometry). The fit to the dispersion relation includes the contributions symmetric and antisymmetric under reversal of the wave vector detailed in Ref. 15 (used parameters for the symmetric part are γ = 28 GHz T^{-1}, A_S =12 pJ/m, M_S = 1.0 T, $\mu_0 H_U$ = 1.15 T, $\mu_0 H_z$ = 0.3 T).

Supplementary Fig. 5b shows only the frequency difference under wave vector reversal $\Delta f = f(k) - f(-k) = 2\gamma/M_S \cdot D_i \cdot k$ which is best suited to extract the interfacial DMI constant D_i. From a fit to the Stokes and Anti-Stokes data, we find D_i = 0.059 ± 0.006 mJ m^{-2} and D_i = 0.049 ± 0.008 mJ m^{-2}, respectively.

Supplementary Fig. 5c shows the linewidth measured at low angles of incidence ($k \approx 0$, ferromagnetic resonance limit) for various magnetic fields between 0.2 T and approx. 0.5 T. From the field-dependent BLS frequency spectra, we extract the resonance frequency and corresponding field linewidth. The comparably large scatter of the data is probably due to the large values of the field linewidth and the resulting instabilities of the setup during the field

sweep. The linewidth is related to the Gilbert damping parameter via:

$$\mu_0 \Delta H = \mu_0 \Delta H_0 + \frac{2\alpha}{\gamma} f \qquad \text{(S10)}$$

From the linear fit to the data, we find $\mu_0 \Delta H_0 \approx 40$ mT, $\alpha = 0.022 \pm 0.05$.

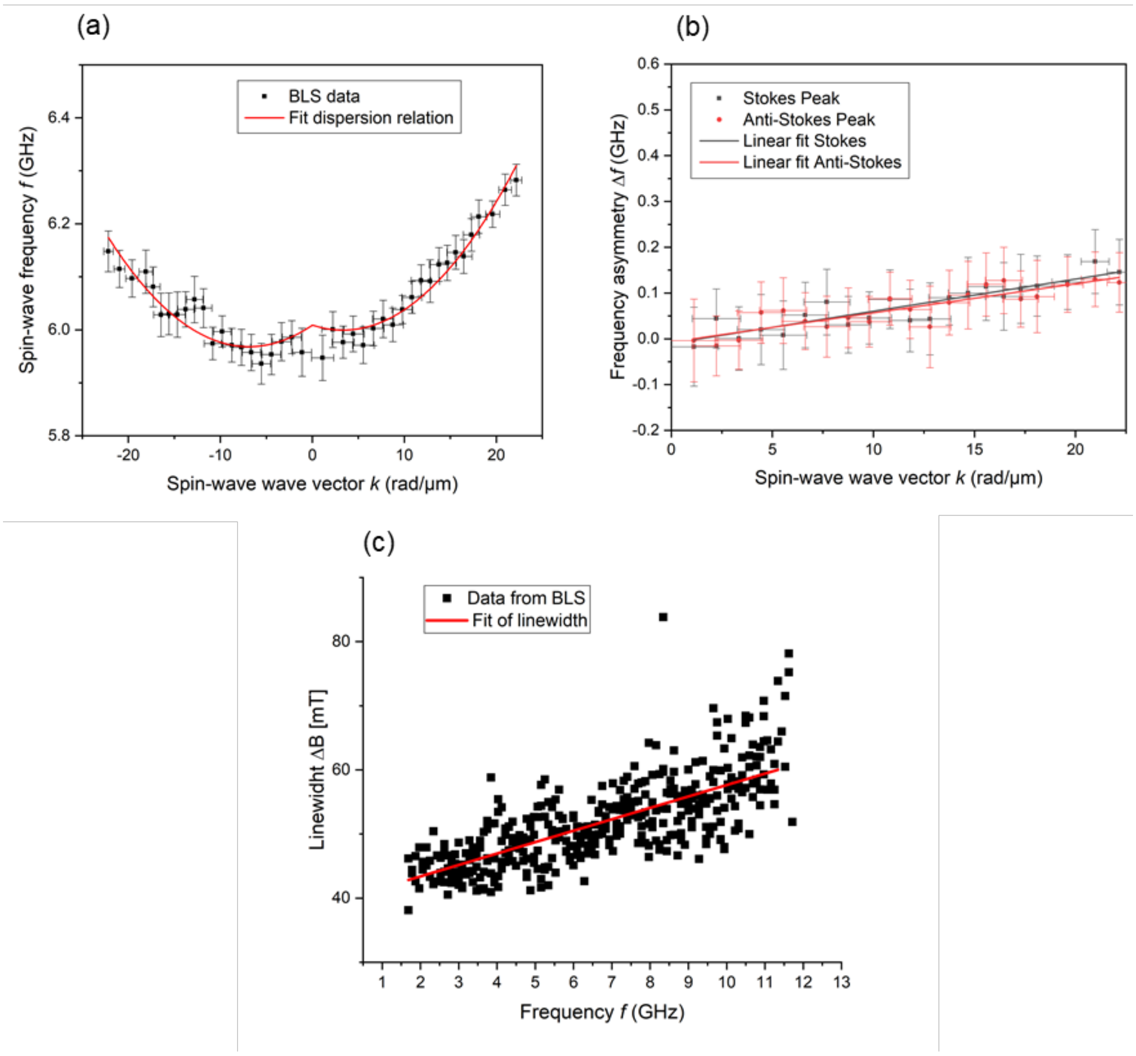

Supplementary Figure 5 | Quantitative evaluation of spin-wave resonances. **a**, The dispersion relation measured at an external field of $\mu_0 H_z = 300$ mT. **b**, The frequency difference under wave vector reversal. **c**, The linewidth of the spin-wave resonances measured at low angles of incidence.

Note 7
Chirality evaluation for bi-layer systems

In order to evaluate the chirality of our SyAFM bi-layer systems, we use micromagnetic simulations with the software MuMax[17]. We show the parameter list used for the simulation in supplementary Table 1, corresponding to the experimental value for the SyAFM system with 90% magnetic compensation. Note that the interlayer magnetic dipole interaction (stray field effect between the top FM and the bottom FM layers) to compete with the interfacial DMI and the interlayer exchange coupling would play a key role in determining the chirality of the system in the case of a relatively small DMI. Thus, we employ the custom fields functionality to incorporate the interlayer exchange coupling field, allowing us to separate the top and bottom FM layers with a finite non-magnetic interlayer[18]. As a consequence, the interlayer magnetic dipole interaction is successfully taken into account in our simulation.

To reveal the effect of interlayer exchange coupling and magnetic compensation on the domain wall (DW) chirality, we make a two-dimensional (2D) phase diagram of the DW angle by varying these parameters. The built-in function "two domain" is used to initialize the domain state for antiparallel configuration between the top and bottom FM layers with the DW at the center. Then, we also use the built-in function "relax" to obtain an equilibrium DW angle.

Supplementary Fig. 6a, b, and c show the DW angle, φ for the up-down DW of the top FM layer, for the down-up DW of the bottom FM layer, and the corresponding domain configuration, respectively. The φ is defined as an azimuthal angle with regard to the x-axis shown in supplementary Fig. 6c. In supplementary Fig. 6a and b, the white circles present the experimental parameter for the stacks used in this work. Concerning the J_{int} dependence, we find the stable AFM coupling DW for the J_{int} roughly larger than 5 μJ m^{-2}. The φ changes with

increasing J_{int} and saturates above some threshold J_{int} depending on the magnetic compensation, m_{Com}. As the experimentally obtained J_{int} is unambiguously larger than the threshold J_{int}, hereafter, we focus on the compensation ratio dependence of φ. Based on the simulation, we can divide the phase into four regions regarding chirality. 1: Left-handed Néel for m_{Com} larger than 90%, 2: Bloch-Néel (left-handed) hybrid for m_{Com} between 90 and 60%, 3: Right-handed Néel for m_{Com} less than 60%, 4. Hybrid Néel for the FM bi-layer system as shown in supplementary Fig. 6c. The interfacial DMI determines the chirality for the highly compensated state simply because both the volume dipole and interlayer dipole interactions are small, even if the DMI magnitude is relatively small. The obtained left-handed chirality for the highly compensated state is consistent with our current-induced collective DW motion. Decreasing m_{Com} gradually increases the contribution of both dipole interactions; thus, the DW chirality gradually changes. With further decreasing m_{Com}, intriguingly, the DW chirality is determined by the interlayer dipole interaction due to the flux closure for domain-domain wall interaction[19] by overcoming the volume dipole contribution, which is a unique feature for the bi-layer system. The chirality is right-handedness in our case (as M_S for the top layer is larger than the bottom counterpart). On top of this, the AFM coupling between DWs induces coherent right-handed chirality for the SyAFM systems. In contrast, the hybrid Néel DW is stabilized for the FM bi-layer system as previously demonstrated[19]. In summary, we demonstrate that the Néel DW is stable in the stacks except for the m_{Com} = 75% case, which would have the Bloch-Néel hybrid DW chirality.

Supplementary Table 1 | Micromagnetic simulation parameter list

World size<br>(top, interlayer, bottom thickness)	512x256x3 nm^3<br>(1 nm, 1 nm,1 nm)
Cell size	2x2x1 nm^3
Periodic boundary condition	32x32x0
Spontaneous magnetization, M_S (top, bottom)	(1.0+x, 1.0-x) T
Effective perpendicular anisotropy field, $\mu_0 H_K^{eff}$	45 mT
Interfacial DMI, D_i	-0.05 mJ m^{-2}
Exchange stiffness, A_S	6 pJ m^{-1}
Interlayer exchange coupling energy J_{int}	x mJ m^{-2}
Gilbert damping, α	1
External perpendicular magnetic field $\mu_0 H_Z$	0.1 mT
Initialization	two domain<br>top: (0, 0, 1, 1, 1, 0, 0, 0.5, -1)<br>bottom: (0, 0, -1, 1, 1, 0, 0, 0.5, 1)

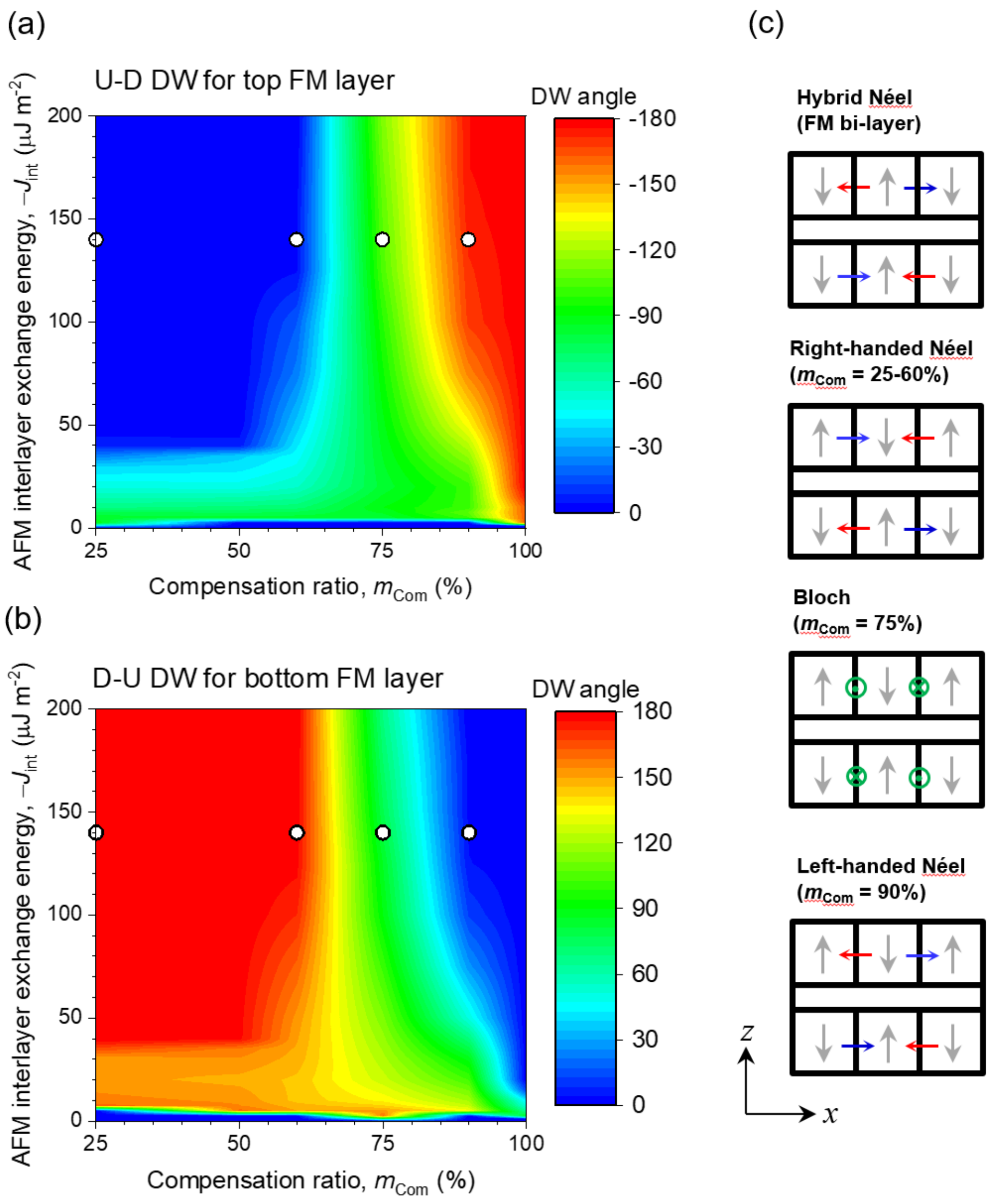

Supplementary Figure 6 | The 2D phase diagram of the domain wall (DW) angle φ defined as an azimuthal angle on the basis of the x-axis. **a**, Up-down DW for a top FM layer. The third and fourth quadrants are used. Red, green, and blue colors correspond to –180, –90, and 0 degrees, respectively. **b**, Down-up DW for a bottom FM layer. The first and second quadrants are used. Red, green, and blue colors correspond to 180, 90, and 0 degrees, respectively. **c**, Domain and DW configuration for various values of the magnetic compensation.

Supplementary References

1. Dohi, T. *et al.* Formation and current-induced motion of synthetic antiferromagnetic skyrmion bubbles. Nat. Commun. **10**, 5153 (2019).
2. Schwieger, S. & Nolting, W. Origin of the temperature dependence of interlayer exchange coupling in metallic trilayers. Phys. Rev. B **69**, 224413 (2004).
3. Schwieger, S. *et al.* Spin-Wave Excitations: The Main Source of the Temperature Dependence of Interlayer Exchange Coupling in Nanostructures. Phys. Rev. Lett. **98**, 057205 (2007).
4. Kalarickal, S. S. *et al.* Dominant role of thermal magnon excitation in temperature dependence of interlayer exchange coupling: Experimental verification. Phys. Rev. B **75**, 224429 (2007).
5. Khodadadi, B. *et al.* Interlayer Exchange Coupling in Asymmetric Co − Fe / Ru / Co − Fe Trilayers Investigated with Broadband Temperature-Dependent Ferromagnetic Resonance. Phys. Rev. Appl. **8**, 014024 (2017).
6. Wang, W. *et al.* Temperature dependence of interlayer exchange coupling and Gilbert damping in synthetic antiferromagnetic trilayers investigated using broadband ferromagnetic resonance. Appl. Phys. Lett. **113**, 042401 (2018).
7. Thiele, A. A. Steady-State Motion of Magnetic Domains. Phys. Rev. Lett. **30**, 230 (1972).
8. Nagaosa, N. & Tokura, Y. Topological properties and dynamics of magnetic skyrmions. Nat. Nanotechnol. **8**, 899 (2013).
9. Schütte, C. *et al.* Inertia, diffusion, and dynamics of a driven skyrmion. Phys. Rev. B **90**, 174434 (2014).
10. Weißenhofer, M. & Nowak, U. Diffusion of skyrmions: the role of topology and anisotropy. New J. Phys. **22**, 103059 (2020).
11. Miltat, J. *et al.* Brownian motion of magnetic domain walls and skyrmions, and their diffusion constants. Phys. Rev. B **97**, 214426 (2018).
12. Weißenhofer, M. *et al.* Skyrmion Dynamics at Finite Temperatures: Beyond Thiele's Equation. Phys. Rev. Lett. **127**, 047203 (2021).

13. Tveten, E. G. *et al.* Staggered Dynamics in Antiferromagnets by Collective Coordinates. Phys. Rev. Lett. **110**, 127208 (2013).

14. Barker, J. & Tretiakov, O. A. Static and Dynamical Properties of Antiferromagnetic Skyrmions in the Presence of Applied Current and Temperature. Phys. Rev. Lett. **116**, 147203 (2016).

15. Böttcher, T. *et al.* Heisenberg Exchange and Dzyaloshinskii–Moriya Interaction in Ultrathin Pt(W)/CoFeB Single and Multilayers. IEEE Trans. Magn. **57**, 1 (2021).

16. Böttcher, T. *et al.* Quantifying symmetric exchange in ultrathin ferromagnetic films with chirality. Phys. Rev. B **107**, 094405 (2023).

17. Vansteenkiste, A. *et al.* The design and verification of MuMax3. AIP Advances **4**, 107133 (2014).

18. Leliaert, J. *et al.* B. MUMAX3-WORKSHOP SESSION 4, 55-64. https://mumax.ugent.be/mumax3-workshop/ (2020).

19. Hrabec, A. *et al.* Current-induced skyrmion generation and dynamics in symmetric bilayers. Nat. Commun. **8**, 15765 (2017).
