# Supplementary material for: Enhanced thermally-activated skyrmion diffusion with tunable effective gyrotropic force
Source: Nat Commun. 2023 Sep 11;14:5424. doi: 10.1038/s41467-023-40720-0 (PMC10495465; doi:10.1038/s41467-023-40720-0)
Supplement: Supplementary file 3 — Description of Additional Supplementary Files [file 41467_2023_40720_MOESM3_ESM.pdf]

Title: Supplementary Movie 1

Description: Skyrmion tracking for 75% magnetic compensation at 320.2 K under magnetic fields of 0.33 mT.

Title: Supplementary Movie 2

Description: Magnetic skyrmions with 90% magnetic compensation at 304.1 K under magnetic fields of 0.06 mT.

Title: Supplementary Movie 3

Description: Magnetic skyrmions with 90% magnetic compensation at 304.7 K under magnetic fields of -0.06 mT.

Title: Supplementary Movie 4

Description: Current-induced collective domain motion using positive current pulse at 302.1 K.

Title: Supplementary Movie 5

Description: Current-induced collective domain motion using negative current pulse at 302.1 K.

Title: Supplementary Movie 6

Description: Atomistic simulation of SyAFM skyrmion diffusion.
